# The correct name for a section of *Ludwigia* L. (Onagraceae)

**DOI:** 10.3897/phytokeys.50.4887

**Published:** 2015-05-27

**Authors:** Peter C. Hoch, Warren L. Wagner, Peter H. Raven

**Affiliations:** 1 Missouri Botanical Garden, P.O. Box 299, St. Louis, MO 63166-0299, USA; 2 Department of Botany, MRC-166, National Museum of Natural History, Smithsonian Institution, P.O. Box 37012, Washington, DC 20013-7012, USA

**Keywords:** *Ludwigia*, *Jussiaea*, sect. *Oligospermum*, nomenclature

## Abstract

In 1953, Hara provided new combinations for many sectional and species names when he combined *Jussiaea* L. with *Ludwigia* L., and at the time, Ludwigia sect. Oligospermum (Micheli) H.Hara was the correct name for one well-defined section. However, subsequent changes to/clarifications of the botanical code have necessitated a change for that name in that now an autonym is treated as having priority over the name or names of the same date and rank that established it. Since Hara’s combination was based on Jussiaea sect. Oligospermum Micheli, the correct name for this section is Ludwigia sect. Jussiaea (L.) Hoch, W.L.Wagner, & P.H.Raven.

## Introduction

The cosmopolitan group of species known since 1953 as Ludwigia sect. Oligospermum is one of the most distinctive in the genus (Raven 1963, Wagner et al. 2007). Among the diplostemonous sections of *Ludwigia* – which formerly were segregated as the genus *Jussiaea* L. – this section differs by having woody, subcylindrical capsules with uniseriate seeds firmly embedded in woody pieces of endosperm and pollen released singly rather than as tetrads or polyads. Most species of the section, which grow in warm-temperate to subtropical moist or wet habitats worldwide, are vigorously aquatic and some (*Ludwigia peploides* (Kunth) P.H.Raven, *Ludwigia hexapetala* (Hook. & Arn.) Zardini, H.Y.Gu & P.H.Raven) can be invasive weeds in wetlands and other wet areas, most recently so in California (Wood 2006, Hoch and Grewell 2012). This polyploid section comprises a group of nine highly variable species that includes three diploid species (n = 8), four tetraploid species (n = 16), one hexaploid species (n = 24, *Ludwigia grandiflora* (Michx.) Greuter & Burdet), and one decaploid species (n = 40, *Ludwigia hexapetala*; see also Nesom and Kartesz 2000). Most species in this section have native distributions restricted to the New World, but two species are restricted to the Old World, *Ludwigia stolonifera* (Guill. & Perr.) P.H.Raven throughout Africa and Madagascar, extending to Turkey and Iraq, and *Ludwigia adscendens* (L.) H.Hara across tropical Asia from India to New Guinea, and from southern Japan to northern Australia, and probably naturalized in Madagascar (Raven 1963, Wagner et al. 2007).

While editing the treatment of *Ludwigia* for the Flora of North America, Jim Zarucchi noticed a problem with the name used for this section, and after consultation with Kanchi Gandhi informed us that a change was necessary. We are grateful to Zarucchi and Gandhi for pointing out this problem for us. We are making this change now so that the correct combination can be available for FNA.

In his treatment for the Flora Brasiliensis (Martius 1875), Micheli divided the genus Jussiaea into three sections: sect. *Eujussiaea*, sect. *Oligospermum*, and sect. *Macrocarpon*. This division of the genus has been widely followed in subsequent treatments. Munz (1942) provided a treatment for New World species of *Jussiaea* in which he recognized the same three sections, but provided different names for two of them (no change for sect. *Macrocarpon* Micheli). For sect. *Eujussiaea* Micheli, he used the new name sect. *Myrtocarpus* Munz, and for sect. *Oligospermum* Micheli, he used sect. *Eujussiaea* Munz. His rationale was that the section that included the type species of the genus had to retain the generic name, and since Hitchcock and Greene (1929) effectively lectotypified *Jussiaea* with *Jussiaea repens* L. [= *Ludwigia adscendens* (L.) Hara], he proposed the name sect. *Eujussiaea* for the section that included *Jussiaea repens* (this lectotypification has been attributed incorrectly in the past to Britton and Brown (1913)).

Hara (1953), following the conclusion by Brenan (1953) and others that *Ludwigia*, *Jussiaea*, and *Isnardia* (a group sometimes segregated) formed a single genus (as *Ludwigia*, as established by Baillon 1877), recognized the sections in question as Ludwigia sect. Oligospermum (Micheli) H.Hara, sect. *Myrtocarpus* (Munz) H.Hara, and sect. *Macrocarpon* (Micheli) H.Hara. He noted that he was “strictly following the Code” (Hara 1953: 290). This treatment was widely accepted, including by Raven (1963) as well as Munz (1965). Most recently Wagner et al. (2007), in a synopsis of Onagraceae, included all three sections as proposed by Hara.

However, changes made to the ICBN in 1981 and retained in subsequent editions (McNeill et al. 2012), specifically as Article 11.6, invalidated part of this treatment. Article 11.6 states that “an autonym is treated as having priority over the name or names of the same date and rank that established it.” So when the transfer of names from *Jussiaea* to *Ludwigia* was made, the correct sectional name combination for Jussiaea sect. Oligospermum should have been Ludwigia sect. Jussiaea, since this is the section that includes the type of the genus. Therefore, we make the following change in compliance with the ICBN.

## Nomenclature

### Ludwigia L. sect. Jussiaea (L.)

Taxon classificationPlantaeMyrtalesOnagraceae

Hoch, W.L.Wagner, & P.H.Raven comb. nov.

urn:lsid:ipni.org:names:77147317-1

Jussiaea L., Sp. pl. 1: 388. 1753. [*Jussia* Adans., Fam. 2: 85, 565. 1763, orth. var.]; Jussiaea L. sect. Eujussiaea Munz, Darwiniana 4: 184. 1944. Type. *Jussiaea repens* L. [=*Ludwigia adscendens* (L.) H.Hara] (Lectotype, designated by Hitchcock & Greene, Prop, Brit. Bot. 153. 1929).
Jussiaea L. sect. Oligospermum Micheli in Martius, Fl. bras. 13(2): 149, 162. 1875. Ludwigia L. sect. Oligospermum (Micheli) H.Hara, J. Jap. Bot. 28: 290. 1953. Type. *Jussiaea hookeri* Micheli [=*Ludwigia hookeri* (Micheli) H.Hara] (Lectotype, designated by Raven, Reinwardtia 6: 335. 1963).
Cubospermum Lour., Fl. Cochinch. 258, 275. 1790. Type. *Cubospermum palustre* (L.) Lour. [= *Ludwigia adscendens* (L.) H.Hara].
Adenola Raf., Aut. Bot. 36. 1840. Type. *Adenola grandiflora* (Michx.) Raf. [= *Ludwigia grandiflora* (Michx.) Greuter & Burdet]. (Lectotype, designated by Pennell, Bull. Torrey Bot. Club 48: 93. 1921).
Oocarpon Micheli, Flora 57: 303. 1874. Ludwigia sect. Oocarpon (Micheli) P.H.Raven, Reinwardtia 6: 336. 1963. Type: *Oocarpon jussiaeoides* Micheli [= *Ludwigia torulosa* (Arn.) H.Hara].

#### Description.

Perennial herbs, stems creeping, floating, or emergent and ascending to erect, rooting at nodes, when floating often forming spongy white pneumatophores at nodes, when erect with spongy base, terete. Leaves alternate; blades with one submarginal vein. Flowers 5(6)-merous; petals present, yellow or white; stamens twice as many (rarely as many) as sepals, pollen shed in monads. Capsules cylindrical, terete, often curved up, woody with thick walls, irregularly and tardily dehiscent. Seeds in one row per locule, pendulous, and firmly embedded in a woody coherent segment of endocarp, with inconspicuous raphe. 2n = 16, 32, 48, 80, 96.

#### Taxa included.

*Ludwigia adscendens* (L.) H.Hara, *Ludwigia grandiflora* (Michx.) Greuter & Burdet, *Ludwigia helminthorrhiza* (Mart.) H.Hara, *Ludwigia hexapetala* (Hook. & Arn.) Zardini, H.Y.Gu & P.H.Raven, *Ludwigia hookeri* (Micheli) H.Hara, *Ludwigia peduncularis* (C.Wright ex Griseb.) M.Gómez, Ludwigia peploides (Kunth) P.H.Raven subsp. glabrescens (O. Kuntze) P.H.Raven, Ludwigia peploides subsp. montevidensis (Sprengel) P.H.Raven, Ludwigia peploides subsp. peploides, Ludwigia peploides subsp. stipulacea (Ohwi) P.H.Raven, *Ludwigia stolonifera* (Guill. & Perr.) P.H.Raven, *Ludwigia torulosa* (Arn.) H.Hara.

## Supplementary Material

XML Treatment for Ludwigia L. sect. Jussiaea (L.)
